# Safeguarding Social Determinants of Health Data at a Managed Health Plan

**DOI:** 10.63116/TLCI7167

**Published:** 2026-02-12

**Authors:** Aurae Beidler, Kevin Rogers

**Affiliations:** School of Healthcare Administration and Leadership, Pacific University

**Keywords:** social determinants of health (SDOH), health equity, healthcare workers (HCWs), data collection, documentation, confidentiality, training, protected health information (PHI), safeguards, patient-centered

## Abstract

**Background:**

Increased adoption of social determinants of health (SDOH) screening protocols by federal and state agencies to address health equity initiatives has led to an increase in the collection of new types of data by health care workers (HCWs). Although there is general consensus on the need for SDOH screening, issues such as a lack of standardization, patient distrust, and concerns over data security continue. Past studies have identified gaps in the methods of SDOH data collection, how the SDOH data is handled, training provided on collecting and safeguarding this type of information, and how to educate patients about how their information may be used. The authors designed this study to explore the perspectives and experiences of HCWs who access and use SDOH data to deliver care coordination for managed health plan members.

**Methods:**

The authors used a mixed-methods methodology, quasi-experimental pre-post study to explore experiences and perspectives regarding data safeguards for SDOH data collected and accessed by HCWs at a managed health plan. Potential participants were selected from 2 divisions that provide care coordination and health-related service needs. The 18-item online survey contained both closed- and open-ended questions, with most using a Likert scale for responses.

**Results:**

The survey response rate was 14.1% (50). The authors found that 78.0% of the respondents were actively accessing and using SDOH data in their role. Of the 39 respondents who actively accessed and used SDOH data in their role, 23 (59.0%) respondents had been trained on how best to collect and use SDOH data, 6 (15.4%) respondents were unsure of receiving training, and 10 (25.6%) respondents had not received training. A majority (33 of 44; 75.0%) of the respondents understood that their department was collecting data directly from individual members regarding their SDOH/health-related social needs. When asked “How certain are you that SDOH data is protected health information (PHI)?,” there was an increase in mean and a decrease in standard deviation values when comparing the pre- and post-study answers to the same question. This indicates a shift toward higher numbers and less variability in results. The results showed that privacy and security training increased the level of confidence that HCWs have in safeguarding SDOH data.

**Conclusions:**

According to the results of this study, HCW participants better understood how SDOH data is protected health information upon receiving training. The results also demonstrated that HCWs are eager and open to more training on safeguarding SDOH data. The findings of this study could be used to assess the current use of SDOH data, develop guidelines for best practices, and identify specific areas of training to ensure the privacy and security of SDOH data.

## BACKGROUND

Nonmedical factors such as housing, food insecurity, and income—termed social determinants of health (SDOH)—can have a bigger influence on health outcomes than health status alone.^1^ The use of screening protocols to collect SDOH data is increasing across the United States owing to widespread awareness of health inequities demonstrated during the COVID-19 pandemic.^2^ As part of its efforts to advance health equity, the Centers for Medicare & Medicaid Services requires SDOH data collection to be conducted using several types of recommended screening protocols.^3^ However, health care workers (HCWs) may not be adequately trained on how to collect this specific type of data, how to transmit and store the data properly, and how to assure patients that their data is private and secure.

Overall, patients and providers agree that SDOH should be screened and addressed.^4,5^ SDOH data helps address health inequities that can be correlated with sex, race or ethnicity, and other social, economic, or geographic markers.^4^ Yet, there is a lack of standardization in SDOH data retrieval and unknown factors related to the use and disclosure of that data, especially race and immigration data points.^6^ HCWs have identified a lack of training on administering SDOH screening tools as a major barrier to SDOH data collection.^7–9^ Owing to a lack of standardization of SDOH screening and data collection, patients and providers may experience redundancies and inefficiencies in the methods for and amount of data collection. Automated systems can alleviate the discomfort of asking redundant questions about sensitive topics, but this approach can also cause harm by way of resurfacing information provided privately to a trusted clinician.^10^

Surveys have shown that patient distrust is a top reason for nonparticipation in SDOH screenings.^11,12^ Patients have expressed concerns about how the data will be used and whether the information will be properly safeguarded and kept confidential.^11^ Patients also may not understand the reason for data collection; they may fear that clinicians will make assumptions and judgments based on the information they provide.^11,13^ Patients want to know the purpose of disclosure and to be assured that the sensitive data being disclosed will not harm them in the long run (eg deportation, stigma, loss of other services, or family dynamics being affected).^11^

A 2020 study^14^ found that patients who were concerned about the security of their medical information were 3 times more likely to withhold information from their provider than patients who were confident in the safeguarding of their information. A recommendation from that study was that providers need to better communicate with patients how their medical information is made secure, as well as how it is used and accessed.^14^

Parents of minors also have reported privacy concerns related to confidentiality of medical records and screening methods.^15^ Some parents expressed that they did not answer questions honestly owing to fear of repercussions, such as reports to family services agencies.^15^ Another reported concern was regarding outdated information, as social needs change frequently.^15^

Because SDOH data is integrated into the clinical record and collected at the time of clinical data capture, SDOH data is treated as protected health information (PHI).^16^ Yet, the concept of treating SDOH as PHI is not broadly understood. There may be a perception that this data type is different than PHI.^17^

There are challenges with integrating sensitive data such as SDOH into electronic health records (EHRs).^13^ Patients in a past study reported that they felt comfortable being asked about their social needs, but they were less comfortable with this data being documented in the EHRs.^17^ Those with more social needs were more concerned about having their information documented in the EHRs.^18^ This is especially true for those who have experienced bias or discrimination.^5^

Training HCWs on how to ensure that SDOH screenings are patient centered is a key to successful data collection.^4^ In a study conducted by the National Opinion Research Center, it was found that although 8 of 10 survey respondents had received training to support the collection and use of SDOH data, especially on privacy and security protections, it may not have been adequate.^19^ Health information security awareness and training affects HCWs’ attitudes toward disclosures of PHI.^20^ If HCWs do not believe that SDOH data provides the same level of confidentiality as PHI, there is uncertainty regarding whether that data will be safeguarded in the same manner.

A previous study of provider perspectives regarding the collection and documentation of gender identity (GI) data found that providers felt uncomfortable asking patients about their GI and that it was not a routine practice; a majority^21^ (76%) of providers expressed a concern that patients may take offense to being asked about GI. Those results demonstrate a need to train health care providers on collecting sensitive data and documenting it, as well as the need for standardized protocols.^21^

## METHODS

### Research Design

In this mixed-methods, quasi-experimental pre-post study, the authors explored participants’ experiences with and perspectives on SDOH. The instrument used in this study was a researcher-created survey. The survey was created owing to lack of existing validated tools. Based on past studies and an author’s (AB) experience with data mapping projects at the health plan, an 18-question survey (Appendix 1) was created. The survey contained both closed- and open-ended questions, with most using a Likert scale for responses. Respondents were queried about previous training, understanding of SDOH data collection, perceptions of privacy and security requirements, and training needs.

The survey tool was created and distributed using the Qualtrics platform (Qualtrics, LLC, Seattle, Washington), in consultation with privacy compliance professionals. A pilot survey was tested for readability and clarity with 10 health care information professionals for a 1-week period in June 2024. These professionals were asked for input on clarity, content, and time to complete the survey. The researchers used the feedback to improve the final version of the survey.

### Ethical Considerations

Before the collection of any data, the research study and the interview questionnaire were submitted to the Pacific University Institutional Review Board for review (075-024). The institutional review board found the study to be exempt.

### Participants and Data Collection

Participants were selected from 2 divisions that provide care coordination and health-related service needs. The survey link was distributed via email by department leaders to 354 employees in total. The survey was open for 1 month, from August 14 to September 14, 2024. Two additional weeks were added to the planned survey time, as the response rate was slower than anticipated.

After the survey closed, the researchers reviewed aggregate data to determine high-level trends. The researchers then met with department leaders to develop a training plan. The training session, titled “Safeguarding Social Determinants of Health Data: Privacy and Security,” was presented during a Care Coordination Department meeting of 150 employees on October 15, 2024. The presentation covered the following topics: a review of SDOH, why privacy matters, treating SDOH as PHI, minimum necessary standards, safeguarding data when receiving data from other organizations, collecting data from members of the health plan, and transmitting data including secure email communication. The presentation was recorded and provided to other departments for viewing. A post-training survey (Appendix 2) was deployed at the end of the training, through Qualtrics, including 7 questions from the original questionnaire.

### Data Analysis

The data analysis was performed using jamovi, an open-source statistical spreadsheet. Data were analyzed using descriptive statistics and 1-sample *t* tests.

## RESULTS

A total of 50 HCWs completed the initial online survey (14.1%; Table 1). Ten (6.7%) of the 150 HCW respondents in attendance at a training session developed based on the original survey results then completed the post-training survey.

**Table 1.** Survey Respondents by Department

Department	n (%) of Respondents
Care Coordination	28 (56.0)
Quality and Health Outcomes	9 (18.0)
Clinical Operations	2 (4.0)
Quality Assurance	2 (4.0)
Social Health	1 (2.0)
Declined	2 (4.0)
Other	6 (12.0)
Total	50

The majority (39; 78.0%) of the respondents to the pre-training survey were actively accessing and using SDOH data in their role (Table 2). Of the 39 respondents who actively access and use SDOH data in their role, 23 (59.0%) had been trained on how best to collect and use SDOH data; the question stated that such training might have contained content on keeping information confidential or how to collect this data in a sensitive manner. Of the remaining respondents, 6 (15.4%) were unsure of receiving training and 10 (25.6%) had not received training.

**Table 2.** Responses Regarding Access and Use of SDOH

Do You Actively Access and Use SDOH Data in Your Role?	n (%) of Respondents
Yes	39 (78.0)
No	11 (22.0)
Total	50

A majority of the respondents (33; 75.0%) said they understood that their department was collecting data directly from individual members regarding their SDOH/health-related social needs (Table 3).

**Table 3.** Responses Regarding Departmental SDOH Data Collection

Does Your Department Collect Data Directly From Individual Members on Their SDOH/Health-Related Social Needs Such As Transportation, Housing, Food Insecurity, or Other?	n (%) of Respondents
Yes	33 (75.0)
No	11 (25.0)
Total	44

The following results were reported in response to the question items on the survey where participants were asked to rank their experience on a Likert scale regarding SDOH as PHI and concerns around safeguarding SDOH data (Tables 4–8). Of the 43 respondents to the question “How certain are you that SDOH data is protected health information (PHI)?,” 29 (67.4%) responded that they were “Very certain or “Somewhat certain” (Table 4). Half of the respondents (22 of 44; 50%) to the question “How concerned are you about the privacy and security of SDOH data?” were “Very concerned” or “Somewhat concerned,” whereas 17 respondents (38.6%) reported being “Somewhat less concerned or Not concerned at all” and 5 (11.4%) were “Indifferent” (Table 5).

**Table 4.** Responses Regarding SDOH as PHI

Survey Question	No. of Respondents	Very Certain n (%)	Somewhat Certain n (%)	Neither Uncertain Nor Certain n (%)	Somewhat Uncertain n (%)	Very Uncertain n (%)
How certain are you that SDOH data is protected health information (PHI)?	43	17 (39.5)	12 (27.9)	7 (16.3)	4 (9.3)	3 (7.0)

**Table 5.** Responses Regarding Concerns About Privacy and Security of SDOH

Survey Question	No. of Respondents	Very Concerned n (%)	Somewhat Concerned n (%)	Indifferent n (%)	Somewhat Less Concerned n (%)	Not Concerned at All n (%)
How concerned are you about the privacy and security of SDOH data?	44	12 (27.3)	10 (22.7)	5 (11.4)	13 (29.5)	4 (9.1)

**Table 6.** Responses Regarding Rating of Members’ Understanding of SDOH Privacy and Security

Survey Question	No. of Respondents	Very Good n (%)	Good n (%)	Neither Poor Nor Good n (%)	Poor n (%)	Very Poor n (%)
How would you rate members’ understanding of the privacy and security of SDOH data?	43	0	5 (11.6)	19 (44.2)	17 (39.5)	2 (4.7)

**Table 7.** Responses Regarding Comfort Addressing Member Concerns About Privacy and Security of SDOH

Survey Question	No. of Respondents	Very Good n (%)	Good n (%)	Neither Poor Nor Good n (%)	Poor n (%)	Very Poor n (%)
How would you rate your comfort level with addressing member privacy and security questions related to their SDOH data?	44	12 (27.3)	14 (31.8)	9 (20.5)	8 (18.2)	1 (2.3)

**Table 8.** Responses Regarding Likelihood of Attending Further Training

Survey Question	No. of Respondents	Very Likely n (%)	Somewhat Likely n (%)	Neither Likely Nor Unlikely n (%)	Somewhat Unlikely n (%)	Very Unlikely n (%)
How likely are you to attend additional training on privacy and security policies of SDOH data?	44	26 (59.1)	12 (27.3)	3 (6.8)	3 (6.8)	0

In response to the question “How would you rate members’ understanding of the privacy and security of SDOH data?,” 19 of the 43 respondents (44.2%) selected “Poor” or “Very poor,” whereas another 19 respondents (11.6%) reported “Neither poor nor good” and only 5 (11.6%) selected “Good” (Table 6).

Responses to the survey question, “How would you rate your comfort level with addressing member privacy and security questions related to their SDOH data?” showed that 26 of the 44 respondents (59.1%) had a “Very good” or “Good” comfort level, whereas 9 respondents (20.5%) reported “Neither poor nor good” and another 9 (20.5%) reported “Poor” or “Very poor” (Table 7).

When asked, “How likely are you to attend additional training on privacy and security policies of SDOH data?,” 38 of the 44 (86.4%) respondents chose “Very likely” or “Somewhat likely,” whereas 3 respondents (6.8%) reported “Neither likely nor unlikely” and another 3 (6.8%) stated “Somewhat unlikely” (Table 8).

When asked, “How certain are you that SDOH data is protected health information (PHI)?,” there was an increase in mean and a decrease in standard deviation values when comparing the pre- and post-study answers to the same question (Table 9). This indicates a shift toward higher numbers and less variability in results.

**Table 9.** Descriptive Statistics for Q12 (Pretraining Survey)/Q3 (Post-Training Survey): “How Certain Are You That SDOH Data Is Protected Health Information (PHI)?”

Quarter	n	Mean	Median	Standard Deviation	Standard Error
Q12	43	3.84	4	1.252	0.191
Q3	6	4.67	5.00	0.516	0.211

Respondents were able to provide write-in answers to 2 open-ended questions about their training needs related to the collection and use of SDOH data as well as how to safeguard SDOH data. The first question on what training would help in the collection and use of SDOH data included responses of training needs on the collection process, general training on SDOH as a whole, how to keep information confidential, treating SDOH like PHI, referrals to community-based organizations (CBOs) and other agencies, documentation in the EHRs, and use of SDOH data for outreach.

The second open-ended question asked what information is needed to help in safeguarding SDOH data, which provided responses on documentation in the electronic record, process-related questions, if SDOH is PHI or included in the Health Insurance Portability and Accountability Act (HIPAA) training, release of information, and partnering with CBOs.

## DISCUSSION

Although the majority of the respondents understood that SDOH is PHI, some did not, and it is important that all HCWs understand the safeguards needed to protect SDOH as PHI. This demonstrates a need for further training in this area. Further research is needed to determine why HCWs may not be concerned about the privacy and security of SDOH data.

Although our HCW survey respondents were somewhat split on their own concern for privacy and security, 19 of 43 (44.2%) the respondents did report that health plan members’ understanding of privacy and security of SDOH was poor or very poor. Our results demonstrate an opportunity for HCWs to connect with health plan members to address their privacy concerns. We also recommend identifying organizational processes and additional training in this area specific to SDOH data privacy and security practices.

The results of this study show that privacy and security training increased the level of confidence that HCWs have in safeguarding SDOH data, which is similar to the findings of previous research.^18^ HCWs better understood that SDOH data is PHI after receiving training. Through the survey responses, HCWs asked to receive more training on safeguarding SDOH data, which also aligns with previous research on training needs.^17^ Ongoing or periodic training could increase HCW awareness and usefulness of training provided.

The qualitative responses suggest a gap in knowledge and HCWs’ confidence surrounding both the technical handling and data safeguards of SDOH data. There is a need for foundational and process-related specific training on SDOH data collection and use, best practices for documenting this data, and addressing member questions on confidentiality of the data collected. The complexity of collaborating with external agencies and CBOs requires tailored training specifically regarding shared standards and data governance practices.

### Limitations

Owing to the difference in sample size between the pretraining and post-training group, a paired *t* test comparison was not feasible. However, a 1-sample *t* test showed an increase in the mean and a decrease in standard deviation values when comparing the pre- and post-study answers to the same question for several Likert scale questions. This indicates a shift toward higher numbers and less variability in results. Further inferential statistics were not possible owing to the low response rate on the post-training survey. Therefore, descriptive results should be interpreted with caution.

There were some study limitations. First, to maintain a high level of confidentiality, we did not link individual responses between pretraining and post-training surveys. Second, our sample size was small. Finally, the researchers are no longer employed by the health plan in which this study was conducted; therefore, it is not possible to apply the conclusions of this data to this specific intervention, although it does inform a starting point for other organizations.

Future research is needed to determine best practices and workflows for SDOH data collection and use.

## CONCLUSIONS

Although more research is needed, the present study’s results suggest that training aimed at improving understanding of privacy and security of SDOH data among HCWs should be created and deployed in a way that empowers HCWs to feel confident in safeguards that secure and protect data. Furthermore, the results provide a deeper understanding of HCWs’ perspectives about the privacy and security of SDOH data, revealing opportunities to improve future training. For vulnerable populations, collection and use of SDOH data may have implications that affect future health services and trust of health care providers. To help ensure proper collection and use of SDOH data, future work is needed to understand patient experiences and best practices for data collection and use.

## DISCLOSURES

The authors have nothing to disclose.

## FUNDING

The authors received no funding for this research.

## Appendix

### APPENDIX 1

Primary Data Collection Tool (Pretraining)

1. Consent
2. What is your department?
  - Behavioral Health
  - Care Coordination
  - Quality and Health Outcomes
  - Clinical Operations
  - Quality Assurance
  - Social Health
  - Other: List
  - Decline to answer
3. Do you actively access and use SDOH data in your role? Social Determinants of Health (SDOH), also referred to as health related social needs, are defined as nonmedical information that impacts health care such as housing, income, food security, and employment information.
  - Yes
  - No
  - I don’t know
4. Have you received training on how best to collect and use SDOH data? Such training might have contained content on keeping information confidential or how to collect this data in a sensitive manner.
  - Yes
  - No
  - I don’t know
5. If you did receive training, who did you receive the training from?
6. How long ago did you receive this training?
  - Less than 1 month ago
  - 1–3 months ago
  - 3–6 months ago
  - 6–12 months ago
  - Over a year ago
  - 1–2 years ago
  - 2 or more years ago
7. How would you rate the training you received?
  - Very poor
  - Poor
  - Neither poor nor good
  - Good
  - Very good
8. Does your department collect data directly from individual members on their SDOH/health-related social needs such as transportation, housing, food insecurity, or other?
  - Yes
  - No
  - I don’t know
  - Other: please specify
9. From which outside sources does your department receive data on members’ SDOH/health-related social needs? Choose all that apply.
  - Health care providers (hospital, clinic)
  - Community/social service organization
  - Social service or community-based referral platform (such as Unite Us)
  - Other: Please specify
  - My department does not receive SDOH data from outside sources.
10. Where is SDOH data stored in your department? Choose all that apply.
  - SharePoint
  - Epic/Compass Rose
  - QNXT
  - iDrive
  - Other: Please specify
  - My department does not store SDOH data.
11. How is SDOH data transmitted or shared outside of your department? Choose all that apply.
  - E-mail
  - Secure portal
  - Epic/CompassRose
  - Fax
  - Telephone
  - Teams chat
  - Teams meeting
  - USPS mail
  - Other: Please specify
  - My department does not transmit or share SDOH data outside our department.
12. How certain are you that SDOH data is protected health information (PHI)? Protected health information is data related to an individual’s present, past, or future health care treatment or payment of treatment defined by the Health Insurance Portability and Accountability Act (HIPAA).
  - Very uncertain
  - Somewhat uncertain
  - Neither uncertain nor certain
  - Somewhat certain
  - Very certain
13. How concerned are you about the privacy and security of SDOH data?
  - Very concerned
  - Somewhat concerned
  - Indifferent
  - Somewhat less concerned
  - Not concerned at all
14. How would you rate members’ understanding of the privacy and security of SDOH data?
  - Very poor
  - Poor
  - Neither poor nor good
  - Good
  - Very poor
15. How would you rate your comfort level with addressing member privacy and security questions related to their SDOH data?
  - Very poor
  - Poor
  - Neither poor nor good
  - Good
  - Very good
16. How likely are you to attend additional training on privacy and security policies of SDOH data?
  - Very unlikely
  - Unlikely
  - Neither unlikely nor likely
  - Somewhat likely
  - Very likely
17. What training would help you in your role related to the collection and use of SDOH data?
18. What information do you need to help you be more successful in safeguarding SDOH data?

### APPENDIX 2

Secondary Data Collection Tool (Post-Training)

Consent

1. Did you complete the SDOH pretraining survey?
  - Yes
  - No
2. Did you attend the live or recorded SDOH training presented to your department?
  - Yes
  - No
3. How certain are you that SDOH data is protected health information (PHI)? Protected health information is data related to an individual’s present, past, or future health care treatment or payment of treatment defined by the Health Insurance Portability and Accountability Act (HIPAA).
  - Very uncertain
  - Somewhat uncertain
  - Neither uncertain nor certain
  - Somewhat certain
  - Very certain
4. How concerned are you about the privacy and security of SDOH data?
  - Very concerned
  - Somewhat concerned
  - Indifferent
  - Somewhat less concerned
  - Not concerned at all
5. How would you rate members’ understanding of the privacy and security of SDOH data?
  - Very poor
  - Poor
  - Neither poor nor good
  - Good
  - Very poor
6. How would you rate your comfort level with addressing member privacy and security questions related to their SDOH data?
  - Very poor
  - Poor
  - Neither poor nor good
  - Good
  - Very good
7. How likely are you to attend additional training on privacy and security policies of SDOH data?
  - Very unlikely
  - Unlikely
  - Neither unlikely nor likely
  - Somewhat likely
  - Very likely
8. What training would help you in your role related to the collection and use of SDOH data?
9. What information do you need to help you be more successful in safeguarding SDOH data?
